# Self-reported COVID-19 infection and implications for mental health and food insecurity among American college students

**DOI:** 10.1073/pnas.2111787119

**Published:** 2022-02-08

**Authors:** Sara Goldrick-Rab, Vanessa Coca, Japbir Gill, Morgan Peele, Kallie Clark, Elizabeth Looker

**Affiliations:** ^a^The Hope Center for College, Community, and Justice, Lewis Katz School of Medicine at Temple University, Philadelphia, PA 19122

**Keywords:** COVID-19, health and well-being, college students, mental health, physical health

## Abstract

While the COVID-19 pandemic affected mental health and increased food insecurity across the general population, less is known about the virus’s impact on college students. A fall 2020 survey of more than 100,000 students at 202 colleges and universities in 42 states reveals sociodemographic variation in self-reported infections, as well as associations between self-reported infection and food insecurity and mental health. We find that 7% of students self-reported a COVID-19 infection, with sizable differences by race/ethnicity, socioeconomic status, parenting status, and student athlete status. Students who self-reported COVID-19 infections were more likely to experience food insecurity, anxiety, and depression. Implications for higher education institutions, policy makers, and students are discussed.

This study examines self-reported COVID-19 infection rates among American college students enrolled during fall 2020 (*n* = 100,488). While some colleges and universities collect information about which of their students contracted COVID-19, the majority do not (1). As college students are returning to classes this fall, this study offers insights into which students have been affected by the disease, and its association with their health and well-being. Conditions brought on by the COVID-19 pandemic are associated with increased anxiety and depressive symptoms (2–5), and food insecurity (6, 7), among college students. The effects of COVID-19 infection, including perceived infection, are much less clear.

In fall 2020, we sent an electronic survey to more than 1.8 million undergraduates enrolled in 202 colleges and universities in 42 states, with a fairly typical response rate of 11% (8, 9). This paper analyzes the results for 100,488 students who responded to questions about whether they had been infected with COVID-19, and how COVID was related to their mental health and food security at the time they took the survey. Given the fact that those most disadvantaged are least likely to respond to surveys, we anticipate the findings here are an underrepresentation of the true infection rates among college students (10). The findings suggest the need for additional, ongoing support for student health and well-being.

## Results

We assessed COVID-19 infection rates by asking students whether or not they were “sick with COVID” at any point during or since the spring 2020 academic term. Incomplete reporting is possible, as some students may have been asymptomatic, unaware they had the virus, or experiencing delayed effects. A study of adults in the United Kingdom found that 24% believed they had the virus but just 4% had tested positive (11). However, there are several reasons why the gap between self-reports and positive tests are likely smaller than reported by the aforementioned study. First, we know that surveys during the pandemic likely underrepresent those most marginalized, as these individuals have lower response rates than those less marginalized (10). Related to this, those who are most marginalized are more likely to have been infected by COVID (12). Finally, as this study examines the association between COVID and mental health, perceptions of infection are equally important, as they are clearly related to mental health and other factors of daily life, given the global recommendations for response upon suspicion of infection (13).

**Fig. 1.**
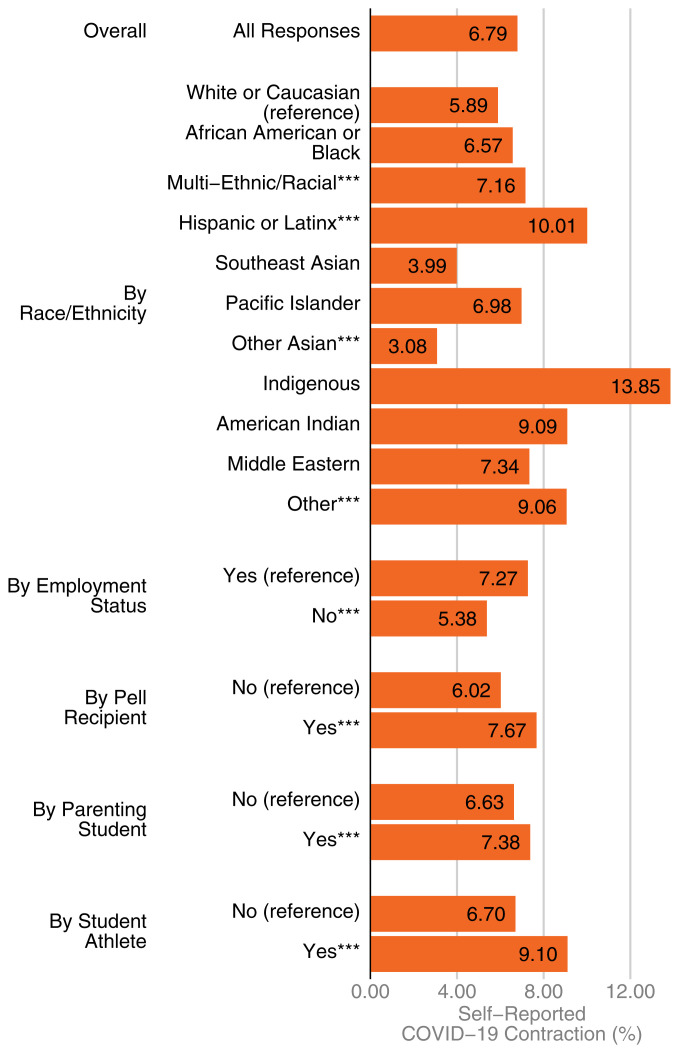
Prevalence of self-reported COVID-19 contraction among college students (*n* = 100,488). Observed rates of self-reports of contracting COVID-19 are sorted by student background characteristics. All subgroups are mutually exclusive. Low socioeconomic status is indicated by whether students received a Pell Grant. * = *P* < 0.05, ** = *P* < 0.01, *** = *P* < 0.001.

Almost 7% of enrolled students (*n* = 6,823; 6.79%) reported that they had COVID-19 (Fig. 1).

Self-reported infection rates were higher among racial/ethnic minorities. For example, 14% of Indigenous students, 10% of Latinx students, and 7% of Black students reported having had the virus, compared to 6% of White students (*P* < 0.01) (Fig. 1). We do not detect differences in self-reported infection rates between female and male students, nor do we find differences based on LGBTQ status, although rates are higher for multigendered students. Students from households of lower socioeconomic standing had substantially higher self-reported rates of infection compared to others (8% vs. 6%, *P* < 0.01).

Several aspects of students’ lives appeared to put them at higher risk of COVID-19 infection. Having children was associated with a higher risk of a self-reported infection (*P* < 0.01). This is not due to age differences between parents and nonparents but might be due to children’s exposure at daycare or school; however, we cannot evaluate those hypotheses with these data. Both working students and college athletes were approximately two percentage points more likely to self-report a COVID-19 infection than nonworking or nonathlete students (*P* < 0.01). For college athletes, it is possible that this difference is not due to heightened risk of infection but rather that student athletes were tested more often and thus more aware of their infection status.

**Fig. 2.**
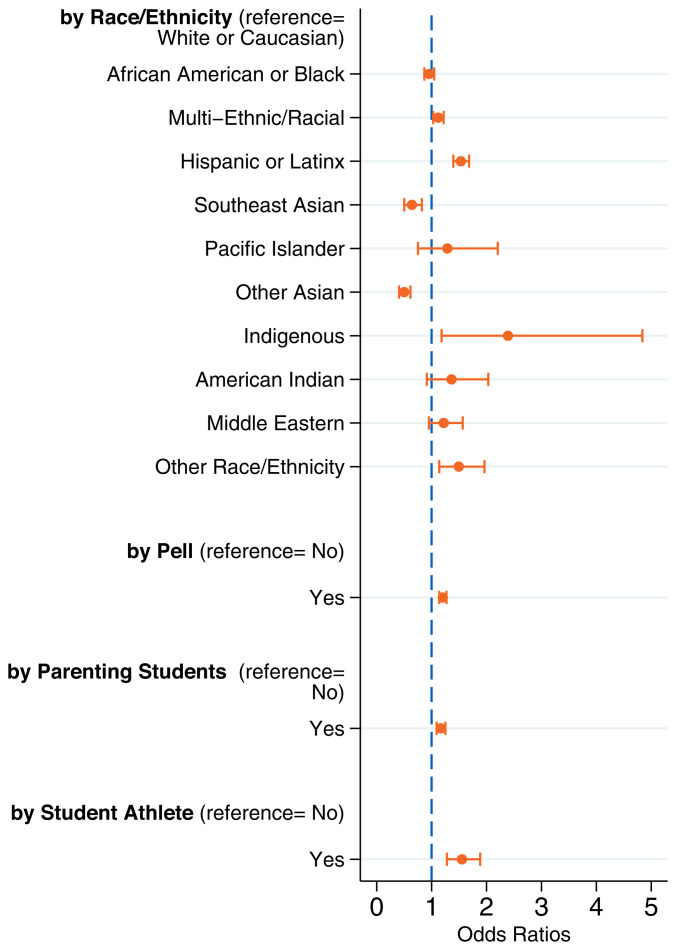
Adjusted odds of self-reported COVID-19 contraction by student characteristics (*n* = 100,488). Odds ratios and 95% CIs for each subgroup are in comparison to the reference group. Low socioeconomic status is indicated by whether students received a Pell Grant.

We implemented multivariate regression analyses to examine whether disparities in reported infection remained after adjusting for the risk factors listed in *SI Appendix*. These adjustments fully accounted for the higher rate of self-reported COVID-19 infection among Black students, but not among Indigenous and Latinx students. Net of observable factors, the odds of self-reported infection were 2.3 times greater (95% CI: 1.2 to 4.8, *P* value: 0.015) for Indigenous students and 1.5 times greater (95% CI: 1.4 to 1.7, *P* value: <0.001) for Latinx students, compared to White students (Fig. 2). Indeed, most of the disparities discussed earlier persist, or even increase, with multivariate modeling.

**Fig. 3.**
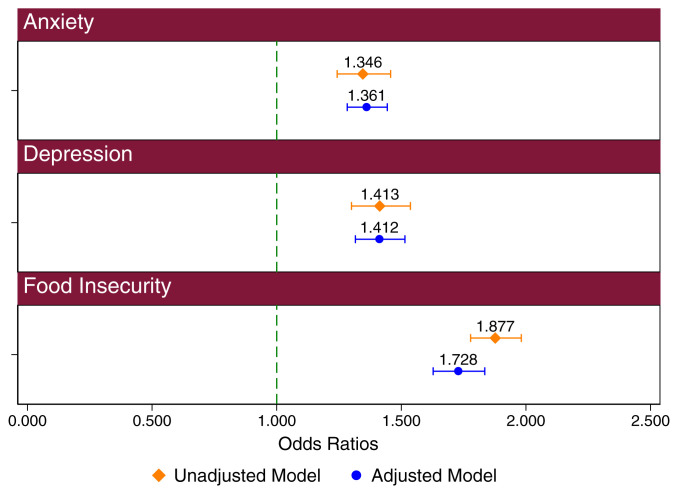
Unadjusted and adjusted odds of anxiety, depression, and food insecurity by self-reported COVID-19 contraction (*n* = 100,488). Unadjusted odds of experiencing anxiety, depression, and food insecurity are compared to adjusted odds. Error bars included in the figure relate to a 95% CI.

We next considered the association between a self-reported COVID-19 infection and mental health and food insecurity as measured by the US Department of Agriculture food security measure. This measure examines multiple factors related to food security based on the ability to maintain consistent, healthy, and affordable access to food. We again used multivariate regression analyses to control for observable differences between students with and without self-reported COVID. Notably, the associations with anxiety, depression, and food insecurity remained nearly the same or increased slightly, after controlling for a range of covariates. Net of observable factors, the odds of experiencing anxiety was 1.4 times greater (95% CI: 1.3 to 1.4, *P* value: <0.001) for a student who self-reported COVID-19 infection than one who did not. Similarly, the odds of experiencing depression were 1.4 times greater (95% CI: 1.3 to 1.5, *P* value: <0.001), while the odds for experiencing food insecurity was 1.7 times greater (95% CI: 1.6 to 1.8, *P* value: <0.001) (Fig. 3).

These findings are consistent with those initially provided by The Hope Center for College Community and Justice (14).

## Discussion

To summarize, this study identifies inequities in self-reported COVID-19 infections among American undergraduates, and negative associations between self-reported COVID-19 infection and depression, anxiety, and food insecurity. With the assumption that our survey’s self-reported COVID-19 infection rate of 7% is generalizable, there could be an estimated 1.4 million college students who have been infected since January 2020. If, as we note above, actual rates of infection exceed self-reports in this survey, then the number of students affected is much larger.

The negative associations between self-reported COVID-19 infection and food insecurity, anxiety, and depression may have several explanations, but new research does suggest increased psychiatric diagnosis after severe COVID-19 infection (15). Given that the longer-term health implications of COVID-19 infections are still being documented (16), it would be prudent for colleges to be prepared to support students who report having been infected with COVID-19. Particular attention should be paid to groups with high rates of self-reported infection, including racially minoritized students, lower-income students, and college athletes.

## Materials and Methods

Data in this study yield from the annual #RealCollege survey fielded in the 2020 fall term at 202 postsecondary colleges and universities across the United States (17). Among the full set of participants in the survey, analyses for this report are from a subset of respondents who had complete information pertaining to whether the student contracted COVID-19, experienced anxiety, experienced depression, experienced food insecurity, and had trouble concentrating. To determine whether significant differences in prevalence of self-reported COVID-19 infection existed across various student subgroups in comparison to specific reference groups, we conducted a series of two-tailed, χ^2^ goodness-of-fit tests with multiple comparison corrections. To estimate differences in self-reported COVID-19 infection by student and institution characteristics and differences in experiences of anxiety, depression, or food insecurity according to whether the student contracted COVID-19, we implemented a series of multivariate logistic regression models run both unconditionally and fully conditionally. Fully conditional models included controls for race and ethnicity, gender, socioeconomic status (SES), parenting status, student age, student athlete status, employment status, learning modality, LGBTQ status, college sector, college regionality, urbanicity, and state.

### Institutional Review Board Approval and Consent.

This study was approved by the Research, Integrity, and Compliance department of the Institutional Review Board at Temple University. A consent form was provided on the first page of the electronic survey such that participants would consent by proceeding through to the survey on the website.

## Supplementary Material

Supplementary File

## Data Availability

Anonymized data, materials, and analysis code are publicly available on the Open Science Framework website (https://osf.io/s4bcv/).
