# Supplementary material for: Self-reported COVID-19 infection and implications for mental health and food insecurity among American college students
Source: Proc Natl Acad Sci U S A. 2022 Feb 8;119(7):e2111787119. doi: 10.1073/pnas.2111787119 (PMC8851491; doi:10.1073/pnas.2111787119)
Supplement: Supplementary File [file pnas.2111787119.sapp.pdf]

Supplementary Information for
Self-Reported COVID-19 Infection and Implications for Mental Health and Food Insecurity among American College Students

Sara Goldrick-Rab,[1] Vanessa Coca,[1]* Japbir Gill,[1] Morgan Peele,[1] Kallie Clark,[1] Elizabeth Looker[1]

[1] The Hope Center for College, Community, and Justice, Lewis Katz School of Medicine at Temple University, Philadelphia, PA

Vanessa Coca
Email: Vanessa.coca@temple.edu

This PDF file includes:

Supplementary text

Other supplementary materials for this manuscript include the following:

Supplementary Information Text

S1.1 Survey Methods

Data in this study yield from an annual survey developed by researchers at the Hope Center for College, Community, and Justice at Temple University, and fielded from the 2020 #RealCollege Survey electronically at 202 colleges and universities across the U.S *(1)*. The Hope Center provided participating colleges with email invitation language and hosted the survey. To reduce sampling bias, language in the email invitation was ambiguous as to the scope of the survey and described the survey as being about "college life".

Upon opening the survey, students were presented with a consent form in compliance with Institutional Review Board standards. To take the survey, the student had to click continue as a record of consent and complete a minimum of the first page of the survey. Participating colleges were asked to use only the provided invitation language to ensure consistency across colleges. In order to boost survey response rates, some colleges also promoted survey participation through text messages and social media. In these cases, they used language and materials provided by the Hope Center.

In 2020, 202 postsecondary colleges and universities fielded the survey early in the fall term, to include students who may cease being enrolled later in the year. Colleges were asked to distribute the survey to all actively enrolled students in the fall of 2020. Response rates are computed as the number of survey participants divided by the number students invited to take the survey. Participating colleges sent survey invitations to an estimated 1.8 million students and 195,629 students participated, yielding an estimated response rate of 10.6%. Participating two-year colleges sent survey invitations to an estimated 1.0 million students, and 112,204 students participated, yielding an estimated response rate of 10.8%. Participating four-year colleges and universities sent survey invitations to an estimated 800,000 students, and 83,425 students participated, yielding an estimated response rate of 10.5%. Respondents attending two-year and four-year colleges had similar mean completion rates of 82 percent.

S1.2 Survey Measures

Self-reported COVID-19 infection rates were assessed through a single survey question, which asked students if they had been "sick with COVID" at any point during or since January 2020 during the COVID-19 pandemic. Responses to the item were limited to either "yes" or "no". Students who answered "yes" were coded "1" for having self-reported COVID-19 contraction and "no" were relatedly coded 0.

To assess food insecurity in the fall of 2020, we used questions from the 18-item Household Food Security Survey Module (shown below) from the U.S. Department of Agriculture (USDA). The 18-item survey includes a subset of questions for students with children.

Food Security Module

Adult Stage 1

1. "In the last 30 days, I worried whether my food would run out before I got money to buy more." (Often true, Sometimes true, Never true)
2. "In the last 30 days, the food that I bought just didn't last, and I didn't have money to get more." (Often true, Sometimes true, Never true)
3. "In the last 30 days, I couldn't afford to eat balanced meals." (Often true, Sometimes true, Never true)

If the respondent answers "often true" or "sometimes true" to any of the three questions in Adult Stage 1, then proceed to Adult Stage 2.

Adult Stage 2

4. "In the last 30 days, did you ever cut the size of your meals or skip meals because there wasn't enough money for food?" (Yes/No)
5. [*If yes to question 4, ask*] "In the last 30 days, how many days did this happen?" (Once, Twice, Three times, Four times, Five times, More than five times)
6. "In the last 30 days, did you ever eat less than you felt you should because there wasn't enough money for food?" (Yes/No)
7. "In the last 30 days, were you ever hungry but didn't eat because there wasn't enough money for food?" (Yes/No)
8. "In the last 30 days, did you lose weight because there wasn't enough money for food?" (Yes/No)

If the respondent answers "yes" to any of the questions in Adult Stage 2, then proceed to Adult Stage 3.

Adult Stage 3

9. "In the last 30 days, did you ever not eat for a whole day because there wasn't enough money for food?" (Yes/No)
10. [*If yes to question 9, ask*] "In the last 30 days, how many days did this happen?" (Once, Twice, Three times, Four times, Five times, More than five times)

If the respondent has indicated that children under 18 are present in the household, then proceed to Child Stage 1.

Child Stage 1

11. “In the last 30 days, I relied on only a few kinds of low-cost food to feed my children because I was running out of money to buy food.” (Often true, Sometimes true, Never true)
12. “In the last 30 days, I couldn’t feed my children a balanced meal, because I couldn’t afford that.” (Often true, Sometimes true, Never true)
13. “In the last 30 days, my child was not eating enough because I just couldn’t afford enough food.” (Often true, Sometimes true, Never true)

If the respondent answers “often true” or “sometimes true” to any of the three questions in Child Stage 1, then proceed to Child Stage 2.

Child Stage 2

14. “In the last 30 days, did you ever cut the size of your children’s meals because there wasn’t enough money for food?” (Yes/No)
15. “In the last 30 days, did your children ever skip meals because there wasn’t enough money for food?” (Yes/No)
16. [*If yes to question 15, ask*] “In the last 30 days, how often did this happen?” (1, 2, 3, 4, 5, 6, 7, 8 or more times)
17. “In the last 30 days, were your children ever hungry but you just couldn’t afford more food?” (Yes/No)
18. “In the last 30 days, did any of your children ever not eat for a whole day because there wasn’t enough money for food?” (Yes/No)

To calculate a raw score for food security, we counted the number of questions to which a student answered affirmatively. “Often true” and “sometimes true” were counted as affirmative answers. Answers of “Three times” or more were counted as a “yes.” Students (with or without children) who had raw scores of zero were considered to have “high” food security. Students (with or without children) with raw scores of one or two were considered to have “marginal” food security. Students without children with raw scores of three to seven were considered to have “low” food security. Students without children with raw scores above eight were considered to have “very low” food security. Students with children with raw scores of three to five were considered to have “low” food security. Students without children with raw scores above five were considered to have “very low” food security. Respondents are considered “food insecure” if they have low or very low levels of food security.

Students’ anxiety levels were assessed using a validated seven-item instrument called the Generalized Anxiety Disorder Scale (GAD-7). In the #RealCollege 2020 survey instrument, the items were separated into two sections (items 1-2 and items 3-7). The student needed to cross a certain score threshold in the first two items to progress to the remaining items. The assessment asked students about the number of times in the last two weeks that they were bothered by any of the following items:

- Feeling nervous, anxious, or on edge;
- Not being able to stop or control worrying;
- Worrying too much about different things;

- Trouble relaxing;
- Being so restless that it's hard to sit still;
- Becoming easily annoyed or irritable;
- Feeling afraid as if something awful might happen.

For each item, students who reported being bothered zero days were coded "0, 1 to 6 days were coded as "1", 7-12 days were coded as "2", and 13-14 days were coded as "3". Item raw scores were then summed. Composite scores of 0 to 4 are categorized as "none to minimal" anxiety. Composite scores of 5 to 9 are categorized as "mild" anxiety. Composite scores of 10 to 14 are categorized as "moderate" anxiety. Composite scores of greater than 14 are categorized as "severe" anxiety. For the purposes of this study, students who were categorized as experiencing "moderate" or "severe" anxiety, are coded as experiencing anxiety, and assigned a dummy value of 1 for anxiety.

Students' depression levels were assessed using a validated nine-item instrument, called the Patient Health Questionnaire (PHQ-9). In the #RealCollege 2020 survey instrument, the items were separated into two sections (items 1-2 and items 3-9). The student needed to reach a certain response threshold in the first two items to progress to the remaining items. The assessment asked students about the number of times in the last two weeks that they were bothered by any of the following items:

- Little interest or pleasure in doing things;
- Feeling down, depressed, or hopeless;
- Trouble falling asleep, staying asleep, or sleeping too much;
- Feeling tired or having little energy; Poor appetite or overeating;
- Feeling bad about yourself—or that you're a failure or have let yourself or your family down;
- Trouble concentrating on things, such as reading the newspaper or watching television;
- Moving or speaking so slowly that other people could have noticed; or the opposite—being so fidgety or restless that you have been moving around a lot more than usual;
- Thought that you would be better off dead or hurting yourself in some way.

For each item, students who reported being bothered zero days were coded "0", 1 to 6 days were coded as "1", 7-12 days were coded as "2", and 13-14 days were coded as "3". Item raw scores were then summed. Composite scores of 0 to 4 are categorized as "none to minimal" depression. Composite scores of 5 to 9 are categorized as "mild" depression. Composite scores of 10 to 14 are categorized as "Moderate" depression. Composite scores of 15 to 19 are categorized as "Moderately Severe" depression. Composite scores of greater than 20 or more are categorized as "Severe" depression. For the purposes of this study, students who were categorized as experiencing "moderate," "moderately severe," or "severe" depression, are coded as experiencing depression, and assigned a dummy value of 1 for depression.

To capture self-reported socioeconomic status (SES) of the household, students were asked whether or not they had received a Pell Grant. Pell Grant eligibility is dependent on income as it is reported on students' Free Application for Federal Student Aid (FAFSA), and all students who wish to receive federal grants and loans must complete a FAFSA to calculate their eligibility. Students who self-reported receiving a Pell Grant were classified as "low SES".

Student's age is self-reported from a single survey item asking for the student's year of birth. The calculation of age is an estimation based on the year the survey was administered: 2020. Student athlete status is assessed by a single survey item asking whether the student is a varsity athlete "on a team sponsored by your college's athletic department". Student parenting status is determined by a single item asking the student if they are "a parent, primary caregiver, or guardian (legal or informal) of any children". Employment status was determined by a single item asking if the student had one job, more than one job, or no job before the COVID-19 pandemic. Students who self-reported having had one job or more than one job were coded to having employment. Learning modality status was assessed through a single item where students answered how they were taking classes in fall 2020 (in-person or online only). All student subgroups are mutually exclusive and derived from survey data. Where student responses had overlapping answers (such as where a student identified with more than one race/ethnicity) an additional "multi" category was created to represent those answers. When student responses are missing information on background information, dummy variables were created and included in analyses where appropriate.

Institutional characteristics, including college sector (two year, four-year), region (West, South, Midwest, Northeast), urbanicity (city, suburb, town, rural), and state are obtained through the National Center for Education Statistics Integrated Postsecondary Education Data System.

S1.3 Analytic Sample

Among the full set of participants from the #RealCollege Survey (N=195,629), analyses conducted for this report are from a subset of respondents who had complete information for questions pertaining to whether the student contracted COVID-19 (N=122,532), experienced anxiety (N=101,080), experienced depression (N=100,894), experienced food insecurity (N=100,803), and had trouble concentrating (N=100,488).

Of our analytic sample (N=100,488), just over half (55%) of sample students were attending two-year colleges, while the rest attended four-year colleges and universities. Racial and ethnic composition was 45% White, 17% Latinx, 10% Black or African American, and 15% multi-ethnic. Two-thirds identified as female, half came from lower socioeconomic households, 20% had children, and 18% identified as LGBTQ. The sample was fairly evenly split by age: 29% were 18-20, 32% were ages 21-25, and 35% were

older than 25. Almost three-quarters were employed, two-thirds lived in a city, 44% were attending at least one in-person class, and just under 2% were student-athletes.

We conducted an additional analysis comparing the survey responses to the study sample and found that students included in the study sample were more likely to complete later stages in the survey, as noticed by the reduced rate of missing answers in many demographic categories which are asked at the end of the survey. This shift in item response mix has significant effects in the comparability of overall survey respondents against the study sample, as noted in the corresponding odds ratios.

Nonetheless, we feel that this does not undermine the generalizability of this study, albeit these results indicate that the study is likely a lower bound estimate of the true rate of COVID

S1.4 Comparison of COVID-19 contraction rates

A series of two-tailed, Chi-square Goodness-of-Fit tests with multiple comparison corrections were conducted to determine if significant differences in prevalence of self-reported COVID-19 infection existed across various student subgroups in comparison to specific reference groups.

S1.5 Estimating the odds of contracting COVID-19

To estimate differences in self-reported COVID-19 infection by student and institution characteristics, we implemented a series of multivariate logistic regression models. The estimates for the likelihood of contracting COVID-19 were run with an unconditional and a fully conditional models. Fully conditional models include controls for race and ethnicity, gender, SES status, parenting student status, student age, student athlete status, employment status, learning modality (in-person or on-line learning), LGTBQ status, college sector, region in which institution is located, urbanicity, and state. Unadjusted and adjusted models incorporate clustered standard errors at the institution level.

S1.6 Estimating odds of experiencing anxiety, depression, or food insecurity

To estimate differences in experiences of anxiety, depression, or food insecurity by whether the student contracted COVID-19, we implemented a series of multivariate logistic regression models. The estimates for the odds of experiencing anxiety, depression, or food insecurity were run with an unconditional and fully conditional model. Fully adjusted models include controls for race and ethnicity, gender, SES status, parenting student status, student age, student athlete status, employment status, learning modality (in-person or on-line learning), LGTBQ status, college sector, region in which institution is located, urbanicity, and state. Unconditional and fully conditional models incorporate clustered standard errors at the institution level.

To check for potential interactions between COVID-19 contraction and certain student characteristics, we ran adjusted models with interaction effects for COVID-19 contraction and factors such as race and ethnicity, SES status, learning modality, employment status, gender, and parenting status. No significant interactions were found that alter the interpretation of the main effects.

S1.7 References

1. S Goldrick-Rab *et al.,* "#RealCollege 2021: Basic Needs Insecurity During the Ongoing Pandemic." https://hope4college.com/rc2021-bni-during-the-ongoing-pandemic/
